# Alpha-Lipoic Acid and Antioxidant Diet Help to Improve Endothelial Dysfunction in Adolescents with Type 1 Diabetes: A Pilot Trial

**DOI:** 10.1155/2015/474561

**Published:** 2015-06-16

**Authors:** Andrea Scaramuzza, Elisa Giani, Francesca Redaelli, Saverio Ungheri, Maddalena Macedoni, Valentina Giudici, Alessandra Bosetti, Matteo Ferrari, Gian Vincenzo Zuccotti

**Affiliations:** ^1^Pediatric Diabetology, Metabolic Diseases & Nutrition, Department of Pediatrics, Azienda Ospedaliera, University of Milano, “Ospedale Luigi Sacco”, 20157 Milan, Italy; ^2^Department of Pediatrics, University of Milano, “Ospedale dei Bambini Vittore Buzzi”, 20154 Milan, Italy

## Abstract

After evaluating the prevalence of early endothelial dysfunction, as measured by means of reactive hyperemia in adolescents with type 1 diabetes, we started a 6-month, double-blind, randomized trial to test the efficacy of an antioxidant diet (± alpha-lipoic acid supplementation) to improve endothelial dysfunction. Seventy-one children and adolescents, ages 17 ± 3.9 yrs, with type 1 diabetes since 9.5 ± 5.3 yrs, using intensified insulin therapy, were randomized into 3 arms: (a) antioxidant diet 10.000 ORAC + alpha-lipoic acid; (b) antioxidant diet 10.000 ORAC + placebo; (c) controls. BMI, blood pressure, fasting lipid profile, HbA1c, insulin requirement, dietary habits, and body composition were determined in each patient. An antioxidant diet significantly improved endothelial dysfunction when supplemented with alpha-lipoic acid, unlike diet with placebo or controls. A significant reduction in bolus insulin was also observed. We speculate that alpha-lipoic acid might have an antioxidant effect in pediatric diabetes patients by reducing insulin.

## 1. Introduction

Atherosclerosis and its vascular damage are the leading cause of mortality and morbidity in patients with diabetes. Recently, several studies have shown that cardiovascular diseases are the first cause of premature death among individuals with type 1 diabetes, demonstrating that although there have been substantial improvements in survival during the past 50 years, still challenges remain [1].

Although diabetes-related vascular complications are uncommon in the pediatric age, early functional and structural abnormalities may be present just a few years after the onset of type 1 diabetes [2]. Therefore, early diagnostic and therapeutic approaches are needed for youth with type 1 diabetes at risk for a premature vascular disease.

A surrogate marker of cardiovascular disease is endothelial dysfunction [3], which is the inability of the artery to sufficiently dilate in response to an appropriate endothelial stimulus. The reactive hyperemia peripheral artery tonometry technique is used for a few years now as a noninvasive test to assess for early vascular changes in high-risk patient groups [4].

In a previous study [5], we evaluated the prevalence of endothelial dysfunction measured as mean of reactive hyperemia index (RHI), in a cohort of adolescents with type 1 diabetes. Surprisingly a low RHI score was observed in 76.7% of patients, showing a dramatic prevalence of early endothelial dysfunction among type 1 diabetes individuals, even in the pediatric age [5].

Endothelial cells have got numerous functions crucial for maintaining intravascular homeostasis: they play an important role in vascular tone regulation, hemostasis, and fibrinolysis and in the production of several substances [6, 7]. In patients with diabetes, hyperglycemia and related pathological biochemical processes trigger damage to the endothelial cells causing their dysfunction. The dysfunctional endothelium adopts prothrombotic, proinflammatory, and vasoconstrictive phenotype promoting the early development of atherosclerosis [3, 6, 7].

Alpha-lipoic acid is a potent mitochondrial antioxidant agent that acts by multiple mechanisms promoting anti-inflammatory and antithrombotic pathways and positively influencing the nitric oxide mediated vasodilatation [8].

In most European countries alpha-lipoic acid is licensed and used as treatment in patients with neuropathic symptoms [9]. Moreover, many studies suggest the potential role of alpha-lipoic acid in prevention and treatment of atherosclerosis and related cardiovascular disease [10, 11].

The hypothesis is that alpha-lipoic acid may improve the endothelial function. This hypothesis is based on similar results obtained either in animal models or in patients with diabetes mellitus, especially type 2 diabetes [12–14]. In streptozotocin-induced rats, it may be assumed that alpha-lipoic acid treatment can protect against impaired vascular responsiveness [12]. In type 2 diabetes patients, alpha-lipoic acid may influence angiogenesis through an effect on some circulating factors including VEGF, bFGF, MCP-1, and IL-10 [14].

Therefore, the aim of our study was to investigate the effect of alpha-lipoic acid on endothelial dysfunction in youth with type 1 diabetes. This pilot study is a 6-month, double-blind, randomized controlled trial to test the efficacy of an antioxidant diet plus alpha-lipoic acid versus an antioxidant diet alone in improving endothelial dysfunction in adolescents with type 1 diabetes.

## 2. Methods

### 2.1. Study Design

This study was a 6-month, prospective, randomized, double-blind, controlled trial conducted in pediatric patients with type 1 diabetes, performed at the Pediatric Department of the University of Milano. The trial was reviewed and approved by the “Luigi Sacco” Hospital Ethics Committee.

The trial was performed in accordance with the Declaration of Helsinki. Before any trial-related activities signed informed consent was obtained from all participants aged 16 years or older and from parents or guardians of participants aged younger than 16 years (assent was obtained from minors).

### 2.2. Participants and Study Procedures

Patients with type 1 diabetes were referred for study participation from the outpatient clinic of the University of Milano between January 2013 and December 2013. Seventy-one patients who had been previously evaluated for the presence of endothelial dysfunction [5] were enrolled in the trial and completed the 6-month study period.

Inclusion criteria were type 1 diabetes (diabetes onset was defined according to ADA criteria), more than 1 year from diagnosis or confirmed C-peptide negative, age between 12 and 19 years, insulin requirement more than or equal to 0.5 U/kg/day, blood glucose checks more the 3 times per day, and insulin intensive therapy ongoing. Exclusion criteria were preexisting cardiovascular diseases or inflammatory systemic diseases, hypertension and prehypertension (BP ≥ 90th percentile for age, sex, and height), eating disorders, obesity (defined as BMI ≥ 95th percentile per age and sex in the 2000 CDC growth chart), celiac disease, inflammatory bowel disease or other significant gastrointestinal conditions, systemic glucocorticoid use (1-month cumulative use during last year) or ongoing treatment with glucocorticoid use, anemia, significant multiple food allergies, uncontrolled hypothyroidism or hyperthyroidism, significant mental illness, and pregnancy.

### 2.3. Measurements

Clinical history and physical examination, nutritional records, and biochemical sample were collected at baseline, after 3 months, and after 6 months; endothelial dysfunction was evaluated at baseline and after 6 months.

### 2.4. Clinical Parameters

Height was measured to the nearest centimeter using a rigid stadiometer. Weight was measured unclothed to the nearest 0.1 kg using a calibrated balance scale. Body mass index (BMI) was calculated for each patient. The pubertal developmental stage was determined according to Marshall and Tanner scale. Blood pressure was measured using a mercury sphygmomanometer, according to the National High Blood Pressure Education Program Working Group. Moreover, 24 h blood pressure was recorded in all patients.

All patients were in intensive insulin therapy, with either multiple daily injections or insulin pump therapy. Daily insulin requirement was calculated as total dose and as basal and bolus rates.

### 2.5. Biochemical Parameters

Blood sampling was performed in the fasting state at 8 a.m. Levels of lipids including triglycerides (TG), total cholesterol (TC), LDL-cholesterol, and HDL-cholesterol were evaluated in all subjects with standard laboratory methods.

Blood was drawn for HbA1c analysis using a fully automated high-performance liquid chromatography system (Variant II, Bio-Rad Laboratories, Munich, Germany).

### 2.6. Nutritional Assessment

At baseline and 3 and 6 months a nutritional visit was performed by the registered dietitian of the Pediatric Department of the “Luigi Sacco” Hospital. Three-day dietary records with nutrition data were evaluated using dedicated software (Metadieta, MeTeDa, San Benedetto del Tronto, AP, Italy). Data on dietary intake, reporting the types and amounts of all food and beverages consumed, with data on insulin doses and algorithms used at home, blood glucose results, and physical activity were collected. During the study period a dietary recall was collected every month; moreover a nutritional assessment was performed at 3 and 6 months to check the patients' compliance to the dietary plan assigned.

Moreover, registered dietitian evaluated the body composition using Tanita BC-418, IL, USA.

### 2.7. Endothelial Dysfunction

Peripheral endothelium-dependent vasodilator capacity was estimated by assessing the RHI by means of the EndoPAT 2000 system (Itamar Medical Ltd., Caesarea, Israel) [5]. Endothelium-mediated changes in vascular tone after occlusion of the brachial artery are reflecting a downstream hyperemic response, which is a measure for arterial endothelial function. The RH-PAT score is calculated as the ratio of the average pulse wave amplitude during 1 min period starting 60 s after cuff deflation divided by the average pulse wave amplitude of a 210 s preocclusion baseline period. Lower RH-PAT scores reflect more pronounced endothelial dysfunction.

### 2.8. Randomization

After the clinical assessment at baseline, each patient was randomly assigned to one of the three double-blind, study arms: 10.000 oxygen radical absorbance capacity units (ORAC) antioxidant diet plus alpha-lipoic acid (*n* = 25, group 1), 10.000 ORAC antioxidant diet plus placebo (*n* = 27, group 2), and controls (*n* = 19, group 3), with no changes in dietary habits. A computer-generated randomization list assigned participants to each group.

Supplementation with 400 mg of slow-release alpha-lipoic acid or placebo, both in a lyophilized formulation, was furnished by the RA of the Pediatric Department. Patients were advised to melt the lyophilized solution in a glass of water and to get it twice a day at least 1 hour before lunch and dinner, for 6 months. The compliance with the antioxidant diet was monitored by monthly dietary recalls and a full nutritional assessment was repeated, after 3 and 6 months.

Both patients treated with antioxidant diet plus alpha-lipoic acid (*n* = 25, group 1) and patients treated with antioxidant diet plus placebo (*n* = 27, group 2) received the same diet plan, of 1800–2200 Kcal per day, according to the age-group. The daily amount of carbohydrates was 60% of the total intake, with 15% of simple carbohydrates. The prescribed amount of fiber was 40 gr per day; fats were 26%, with a monosaturated percentage of 12, polysaturated percentage of 4, and saturated percentage of 8. The ratio of the fatty acid was 8 gr for omega-6 and 2 gr for omega-3. The daily amount of proteins was 14% of the total intake, with 54% of animal proteins and 46% of vegetable proteins. Moreover at least 10.000 ORAC as foods were prescribed in the dietary plan. No particular nutrient source to elevate the dietary ORAC was administered, but a list of foods with high ORAC content was given to each subject with detailed instruction to reach the desired assumption of ORAC.

A similar dietary plan was assigned to patients enrolled in the control group (*n* = 19, group 3), but no ORAC supplementation was suggested.

### 2.9. Statistics

Prior to data analysis, all metric variables were checked for normality by using Shapiro-Wilk test. Data were normally distributed. We used unpaired *t*-test and MANOVA (multiple analysis of variance) when appropriate. Results are presented as mean ± standard deviation (SD), or percentage, unless stated otherwise. All *p* values less than 0.05 were considered statistically significant. Statistical analyses were performed using IBM SPSS Statistics package version 20.

## 3. Results

All the 71 patients enrolled completed the 6-month study period and no one dropped out of study before completing it. Moreover no severe side effects have been reported by either alpha-lipoic acid group or placebo. Either severe hypoglycemic events or DKA episodes occurred during the 6-month study. Three patients of the alpha-lipoic acid group and 2 patients of the control group reported mild abdominal pain over the first 4 weeks of the study, with no need to withdraw from the study.

Baseline and 6-month clinical characteristics are summarized in Table 1 for each study group. At baseline, the mean age of the whole group was 16.3 ± 3.4 years, the mean of diabetes duration was 8.1 ± 5.2 years, the mean BMI was 21 ± 3, and insulin requirement was 0.83 ± 0.29 U/kg/day. The mean value of HbA1c (%) was 7.96 ± 1.04 at baseline. All patients enrolled in the study reported in-range levels of triglycerides, total cholesterol, LDL-cholesterol, and HDL-cholesterol per age and sex over the study period. Moreover all patients showed normal blood pressure measurements per age, sex, and height. There was no statistical difference in all the demographic and clinical variables considered among the three study groups at baseline (Table 1). After 3 months, a significant reduction in daily insulin requirements was observed only in group 1, both in the amount of the total daily dose (0.74 U/kg/day at 3 months versus 0.83 U/kg/day at baseline, *p* = 0.048) and in the percentage of bolus (22.0% at 3 months versus 26.3% at baseline, *p* = 0.047) (Table 1). However these data were not confirmed at 6 months, and no differences were reported among the 3 groups after 6 months of diet in terms of daily insulin requirement.

These results are supported by data collected during the nutritional assessment. Indeed, dietary recall to evaluate patients' compliance to the dietary plan showed a significant increase of ORAC intake at month 3 in both arms (treatment and placebo) but not in controls. After 6 months, ORAC consumption decreased in the treatment group but not in the placebo group, as reported in Table 1.

Endothelial dysfunction was tested as RHI score in all groups at baseline and after 6 months.

**Figure 1:**
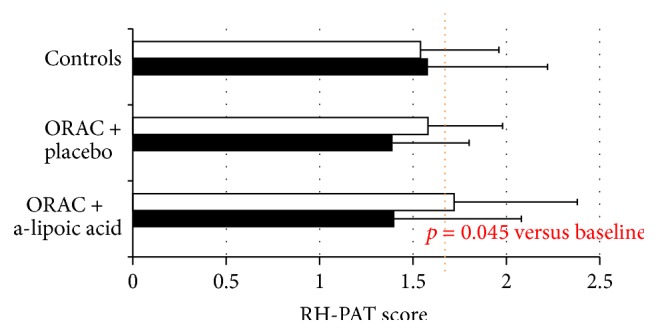
Endothelial function as RH-PAT score at baseline (■) and after 6-month follow-up (□); dotted line represents the normal value for the test.

As described in a previous study [5], at baseline a low RHI score was reported in all patients enrolled, which resulted in a pathologic range (<1.67). At 6 months, RHI score significantly improved only in the group of patients treated with antioxidant diet plus alpha-lipoic acid (1.72 versus 1.4, *p* = 0.045), reaching a normal RHI value [5] (Table 1 and Figure 1). The group of patients treated with antioxidant diet plus placebo reported a better RHI score at 6 months versus baseline as well, but it did not reach the significance. No differences in RHI score were showed in the control group at 6 months versus baseline.

The analyses performed on the other clinical parameters collected showed no differences at baseline and after 3 and 6 months among the three groups: BMI, 24 h blood pressure, lipid profile, HbA1c, dietary habits, and body composition resulted any different among the three groups and over the study period.

## 4. Discussion and Conclusion

To the best of our knowledge, this is the first study analyzing the effects of a dietary supplementation in improving the endothelial dysfunction in youth with type 1 diabetes.

In this randomized, placebo controlled double-blind trial, we found a relationship between the consumption of an antioxidant diet plus alpha-lipoic acid and the improvement in endothelial dysfunction in youth with type 1 diabetes. In particular an antioxidant diet (10000 ORAC) plus alpha-lipoic acid (800 mg/day) seems to be effective in reducing insulin requirement and daily bolus rate after 3 months and in improving endothelial dysfunction after 6 months.

The efficacy of alpha-lipoic acid in improving glucose disposal has been previously described in animal models [12–15] and in type 2 diabetes adult patients [13, 14, 16]. It was 1970 when lipoic acid was shown to enhance glucose uptake into rat tissue [17, 18]. Gradually the effects of different formulas of alpha-lipoic acid were tested both in vitro and in animal models [16, 19] confirming the ability to increase glucose uptake and to enhance the glycogen synthesis [16, 19]. The molecule seems to work improving insulin sensitivity by modulating the signal transduction and by increasing the glucose uptake. In patients with type 2 diabetes, an acute intravenous administration of 1000 mg of lipoic acid was reported to significantly improve insulin-stimulated glucose disposal [20]. Moreover a 4-week oral treatment with alpha-lipoic acid successfully resulted in increasing insulin sensitivity in patients with type 2 diabetes [21].

In our small study, a supplementation with 800 mg/day of alpha-lipoic acid for 3 months effectively resulted in reducing insulin requirement. The mechanisms of action are not completely clear, since, in our knowledge, studies having pediatric type 1 diabetes patients as target are lacking. However, as reported for type 2 diabetes, we hypothesize that alpha-lipoic acid can use nicotinamide adenine dinucleotide molecules (NADH) for the reduction to dihydrolipoic acid, resulting in an increased ratio of nicotinamide adenine dinucleotide (NAD+) to NADH and thus stimulating the glycolysis pathway [22].

In our study the alpha-lipoic beneficial effect on insulin requirement and thus on glucose uptake is reported at 3 months but it is not confirmed at 6 months. In our opinion this can be explained by a decreased compliance to the dietary supplementation, as confirmed by dietary recall and by data collected during the nutritional assessments.

We also demonstrated for the very first time the power of alpha-lipoic acid in improving endothelial dysfunction in youths with type 1 diabetes. In our cohort of patients, the prevalence of endothelial dysfunction is largely represented, despite the pediatric age and the quite good metabolic control [5]. Data from registries has recently shown that a large proportion of the type 1 diabetic population does not meet the age-associated HbA1c targets across all countries, especially in the youth age [23]. Moreover, data recently published by Lind and colleagues [24] showed that patients with type 1 diabetes and on-target HbA1c still have a risk of death from any cause and from cardiovascular diseases more than twice the risks in the general population. These dramatic data ask for new insights into the pathogenesis of diabetes-related complications, as well as for new therapeutic approaches.

Thus, during the last decades, many studies have been conducted to investigate the role of inflammation in diabetes onset and in diabetes-related complications. The opportunity for an early detection of endothelial dysfunction can lead to new insights into the very first steps of the cardiovascular complications. Indeed, endothelial cells play a wide spectrum of different functions, as regulating coagulation, leukocyte adhesion, and trafficking, modifying the tone of the vessel, and participating in the smooth-muscle growth [22]. As a consequence, many different pathways can be affected by pathologic factors, leading to endothelial dysfunction. Several mechanisms have been suggested to explain endothelial dysfunction: hyperglycemia itself [25], increased oxidative stress and subsequent AGEs production and protein kinase C and polyol-pathway activation [26], lower vitamin C plasma concentration, and significantly higher circulating reactive protein C levels [27, 28].

Alpha-lipoic acid is reported to be effective in most of these reactions, getting the role of antioxidant, anti-inflammatory molecule. Indeed, although it is well defined as a therapy for preventing diabetic polyneuropathies and it is used in the management of diabetic peripheral neuropathy in both patients with type 1 and type 2 diabetes, alpha-lipoic acid has many biochemical functions. It acts as metal chelator, as scavenger of free radicals, and as reducer of the oxidized forms of other antioxidant agents such as vitamins C and E and it regulates several signal transductions [14]. In particular alpha-lipoic acid was shown to increase the activity of eNOS, to downregulate the expression of the cell-adhesion molecules ICAM-1 and VCAM-1 and of MMP-9 by the inhibition of AGE-induced NF-kB (nuclear factor kappa-light-chain-enhancer of activated B cells) adhesion factors [14, 22].

Supported by this evidence, we can speculate that a 6-month supplementation with alpha-lipoic acid 800 mg/day can positively impact endothelial dysfunction, decreasing oxidative stress and inflammation in type 1 diabetes, even in pediatrics.

Despite this, these data must be carefully weighed against the lack of consensus regarding the most appropriate supplementation method for alpha-lipoic acid, dosage, and treatment duration.

However, in a paper recently published a 3-month treatment with 600 mg of alpha-lipoic acid was demonstrated to provide significant beneficial changes in VEGF, bFGF, MCO-1, and IL-10 serum levels in type 2 diabetic patients, confirming the efficacy of low-period treatment on endothelial outcomes [29]. Moreover, several studies demonstrated the efficacy of 600 or more mg of alpha-lipoic acid administered for few weeks or months [30, 31] in the treatment of diabetic polyneuropathy.

The strength of our study is the evidence for the first time of a positive association between alpha-lipoic administration and decreased endothelial dysfunction in pediatric patients with type 1 diabetes. Nevertheless the trial has some limitations, and we strongly advise a larger sample and a long-term follow-up to confirm these results. Moreover, the effects of alpha-lipoic acid on glycemic control need to be investigated as well, both as glycemic variability and hypo- and hyperglycemic events.

## Figures and Tables

**Table 1:** Clinical characteristics of the three study groups at baseline and after 3 and 6 months of follow-up.

	Group 1 (*n* = 25)	Group 2 (*n* = 27)	Group 3 (*n* = 19)	All (*n* = 71)	*p* ^*∗*^
Sex (M/F)	15/10	16/11	11/8	42/29	0.699

Age (years)	16.1 ± 3.1	16.0 ± 3.4	16.5 ± 4.3	16.3 ± 3.4	0.916

Diabetes duration (years)	7.7 ± 4.9	8.2 ± 5.6	8.8 ± 5.7	8.1 ± 5.2	0.743

BMI (kg/m^2^)	21 ± 2	22 ± 3	21 ± 3	21 ± 3	0.911

Insulin requirement (U/kg/day)	0.83 ± 0.26	0.85 ± 0.31	0.77 ± 0.33	0.83 ± 0.29	0.775

Insulin requirement (U/kg/day) after 3 months	0.74 ± 0.18 **0.049 versus baseline**	0.86 ± 0.27	0.86 ± 0.19	0.82 ± 0.21	0.211

Insulin requirement (U/kg/day) after 6 months	0.85 ± 0.30 **0.007 versus 3 mo**	0.83 ± 0.29	0.89 ± 0.19	0.86 ± 0.26	0.887

Basal dose (U/day - %)	25.5 ± 8.6 (50 ± 11)	25.4 ± 7.6 (51 ± 9)	28.4 ± 12.2 (50 ± 11)	26.4 ± 9.5 (50 ± 10)	0.733 0.789

Basal dose (U/day - %) after 3 months	25.9 ± 9.4 (53 ± 12)	25.4 ± 8.0 (51 ± 7)	29.4 ± 10.7 (52 ± 8)	27.0 ± 9.4 (52 ± 9)	0.513 0.563

Basal dose (U/day - %) after 6 months	25.8 ± 9.4 (52 ± 14)	25.6 ± 8.3 (52 ± 9)	29.6 ± 11.3 (52 ± 9)	27.2 ± 9.7 (59 ± 10)	0.513 0.996

Bolus dose (U/day - %)	26.3 ± 10.8 (50 ± 12)	25.6 ± 13.2 (51 ± 9)	24.4 ± 9.0 (47 ± 7)	25.4 ± 11.0 (49 ± 9)	0.916 0.739

Bolus dose (U/day - %) after 3 months	22.0 ± 9.4 (47 ± 12) **0.047 versus baseline**	25.8 ± 11.9 (49 ± 7)	25.9 ± 6.4 (48 ± 8)	24.6 ± 9.2 (48 ± 9)	0.401 0.564

Bolus dose (U/day - %) after 6 months	25.9 ± 13.3 (48 ± 14)	24.1 ± 12.4 (47 ± 9)	25.9 ± 6.1 (48 ± 9)	25.3 ± 10.6 (48 ± 11)	0.864 0.989

HbA1c (%)	7.97 ± 1.19	7.96 ± 1.04	7.91 ± 0.76	7.96 ± 1.04	0.856

Reactive hyperemia index	1.40 ± 0.68	1.39 ± 0.41	1.58 ± 0.64	1.47 ± 0.59	0.349

Reactive hyperemia index after 6 months	1.72 ± 0.66 **0.045 versus baseline**	1.58 ± 0.40	1.54 ± 0.42	1.62 ± 0.51	**0.002**

ORAC in the diet	8872,43 ± 5378,22	9771,96 ± 6266,26	9503,25 ± 9973,67	9382,55 ± 7206,05	0.638

ORAC in the diet at 3 months	11844,09 ± 5603,01	12874,04 ± 7165,52	8489,71 ± 6946,57	11069,28 ± 6571,53	**0.036**

ORAC in the diet at 6 months	12518,70 ± 5908,64	15496,83 ± 6897,98	11306,83 ± 11737,31	13107,45 ± 8181,31	0.098

^*∗*^By MANOVA test.

BMI = body mass index; HbA1c = glycosylated hemoglobin; ORAC = oxygen radical absorbance capacity.

## References

[1] Livingstone S. J., Levin D., Looker H. C. (2015). Estimated life expectancy in a Scottish cohort with type 1 diabetes, 2008–2010. *The Journal of the American Medical Association*.

[2] Dalla Pozza R., Bechtold S., Bonfig W. (2007). Age of onset of type 1 diabetes in children and carotid intima medial thickness. *The Journal of Clinical Endocrinology & Metabolism*.

[3] Widlansky M. E., Gokce N., Keaney J. F., Vita J. A. (2003). The clinical implications of endothelial dysfunction. *Journal of the American College of Cardiology*.

[4] Pareyn A., Allegaert K., Asscherickx W., Peirsman E., Verhamme P., Casteels K. (2013). Impaired endothelial function in female adolescents with type 1 diabetes measured by peripheral artery tonometry. *European Journal of Pediatrics*.

[5] Scaramuzza A. E., Redaelli F., Giani E. (2015). Adolescents and young adults with type 1 diabetes display a high prevalence of endothelial dysfunction. *Acta Paediatrica*.

[6] Bakker W., Eringa E. C., Sipkema P., van Hinsbergh V. W. M. (2009). Endothelial dysfunction and diabetes: roles of hyperglycemia, impaired insulin signaling and obesity. *Cell and Tissue Research*.

[7] Schalkwijk C. G., Stehouwer C. D. A. (2005). Vascular complications in diabetes mellitus: the role of endothelial dysfunction. *Clinical Science*.

[8] Heitzer T., Finckh B., Albers S., Krohn K., Kohlschütter A., Meinertz T. (2001). Beneficial effects of *α*-lipoic acid and ascorbic acid on endothelium-dependent, nitric oxide-mediated vasodilation in diabetic patients: relation to parameters of oxidative stress. *Free Radical Biology and Medicine*.

[9] Boulton A. J. M., Kempler P., Ametov A., Ziegler D. (2013). Whither pathogenetic treatments for diabetic polyneuropathy?. *Diabetes/Metabolism Research and Reviews*.

[10] Da Ros R., Assaloni R., Ceriello A. (2005). Molecular targets of diabetic vascular complications and potential new drugs. *Current Drug Targets*.

[11] Ying Z., Kherada N., Farrar B. (2010). Lipoic acid effects on established atherosclerosis. *Life Sciences*.

[12] Okudan N., Nurullahoğlu Atalik K. E., Gökbel H., Canbilen A., Kara I. (2011). Alpha lipoic acid treatment improved endothelium-dependent relaxation in diabetic rat aorta. *Yakugaku Zasshi*.

[13] Coletta C., Módis K., Szczesny B. (2015). Regulation of vascular tone, angiogenesis and cellular bioenergetics by the 3-mercaptopyruvate sulfurtransferase/H_2_S pathway: functional impairment by hyperglycemia and restoration by dl-*α*-lipoic acid. *Molecular Medicine*.

[14] Dworacka M., Iskakova S., Krzyżagórska E., Wesołowska A., Kurmambayev Y., Dworacki G. (2015). Alpha-lipoic acid modifies circulating angiogenic factors in patients with type 2 diabetes mellitus. *Diabetes Research and Clinical Practice*.

[15] Bonetti P. O., Pumper G. M., Higano S. T., Holmes D. R., Kuvin J. T., Lerman A. (2004). Noninvasive identification of patients with early coronary atherosclerosis by assessment of digital reactive hyperemia. *Journal of the American College of Cardiology*.

[16] Jacob S., Streeper R. S., Fogt D. L. (1996). The antioxidant *α*-lipoic acid enhances insulin-stimulated glucose metabolism in insulin-resistant rat skeletal muscle. *Diabetes*.

[17] Haugaard N., Haugaard E. S. (1970). Stimulation of glucose utilization by thioctic acid in rat diaphragm incubated in vitro. *Biochimica et Biophysica Acta—General Subjects*.

[18] Singh H. P. P., Bowman R. H. (1970). Effect of DL-*α*-lipoic acid on the citrate concentration and phosphofructokinase activity of perfused hearts from normal and diabetic rats. *Biochemical and Biophysical Research Communications*.

[19] Streeper R. S., Henriksen E. J., Jacob S., Hokama J. Y., Fogt D. L., Tritschler H. J. (1997). Differential effects of lipoic acid stereoisomers on glucose metabolism in insulin-resistant skeletal muscle. *American Journal of Physiology—Endocrinology and Metabolism*.

[20] Jacob S., Henriksen E. J., Schiemann A. L. (1995). Enhancement of glucose disposal in patients with type 2 diabetes by alpha-lipoic acid. *Arzneimittel-Forschung*.

[21] Jacob S., Ruus P., Hermann R. (1999). Oral administration of RAC-*α*-lipoic acid modulates insulin sensitivity in patients with type-2 diabetes mellitus: a placebo-controlled pilot trial. *Free Radical Biology and Medicine*.

[22] Packer L., Kraemer K., Rimbach G. (2001). Molecular aspects of lipoic acid in the prevention of diabetes complications. *Nutrition*.

[23] McKnight J. A., Wild S. H., Lamb M. J. (2015). Glycaemic control of Type 1 diabetes in clinical practice early in the 21st century: an international comparison. *Diabetic Medicine*.

[24] Lind M., Svensson A., Kosiborod M. (2014). Glycemic control and excess mortality in type 1 diabetes. *The New England Journal of Medicine*.

[25] Haller M. J., Stein J., Shuster J. (2007). Peripheral artery tonometry demonstrates altered endothelial function in children with type 1 diabetes. *Pediatric Diabetes*.

[26] Järvisalo M. J., Raitakari M., Toikka J. O. (2004). Endothelial dysfunction and increased arterial intima media thickness in children with type 1 diabetes. *Circulation*.

[27] King G. L., Loeken M. R. (2004). Hyperglycemia-induced oxidative stress in diabetic complications. *Histochemistry and Cell Biology*.

[28] Gomes M. B., Negrato C. A. (2014). Alpha-lipoic acid as a pleiotropic compound with potential therapeutic use in diabetes and other chronic diseases. *Diabetology & Metabolic Syndrome*.

[29] Haak E. S., Usadel K. H., Kohleisen M., Yilmaz A., Kusterer K., Haak T. (1999). The effect of *α*-lipoic acid on the neurovascular reflex arc in patients with diabetic neuropathy assessed by capillary microscopy. *Microvascular Research*.

[30] Ziegler D., Gries F. A. (1997). *α*-lipoic acid in the treatment of diabetic peripheral and cardiac autonomic neuropathy. *Diabetes*.

[31] Eltayeb A. A., Ahmad F.-A., Sayed D. M., Osama A. M. (2014). Subclinical vascular endothelial dysfunctions and myocardial changes with type 1 diabetes mellitus in children and adolescents. *Pediatric Cardiology*.

